# Paclitaxel Modulates TGFβ Signaling in Scleroderma Skin Grafts in Immunodeficient Mice

**DOI:** 10.1371/journal.pmed.0020354

**Published:** 2005-11-01

**Authors:** Xialin Liu, Shoukang Zhu, Tao Wang, Laura Hummers, Fredrick M Wigley, Pascal J Goldschmidt-Clermont, Chunming Dong

**Affiliations:** 1Division of Cardiology, Department of Medicine, Duke University Medical Center, Durham, North Carolina, United States of America,; **2**Division of Rheumatology, Johns Hopkins University, Baltimore, Maryland, United States of America; Leiden University Medical CentreThe Netherlands

## Abstract

**Background:**

Systemic sclerosis (SSc) is characterized by excessive fibrosis and obliterative vascular lesions. Abnormal TGFβ activation is implicated in the pathogenesis of SSc. Aberrant TGFβ/Smad signaling can be controlled by stabilization of microtubules with paclitaxel.

**Methods and Findings:**

SSc and healthy human skin biopsies were incubated in the presence or absence of paclitaxel followed by transplantation into severe combined immunodeficient mice. TGFβ signaling, fibrosis, and neovessel formation were evaluated by quantitative RT-PCR and immunohistochemical staining. Paclitaxel markedly suppressed Smad2 and Smad3 phosphorylation and collagen deposition in SSc grafts. As a result, the autonomous maintenance/reconstitution of the SSc phenotype was prevented. Remarkably, SSc grafts showed a 2-fold increase in neovessel formation relative to normal grafts, regardless of paclitaxel treatment. Angiogenesis in SSc grafts was associated with a substantial increase in mouse PECAM-1 expression, indicating the mouse origin of the neovascular cells.

**Conclusion:**

Low-dose paclitaxel can significantly suppress TGFβ/Smad activity and lessen fibrosis in SCID mice. Transplantation of SSc skin into SCID mice elicits a strong angiogenesis—an effect not affected by paclitaxel. Although prolonged chemotherapy with paclitaxel at higher doses is associated with pro-fibrotic and anti-angiogenic changes, the findings described here indicate that low-dose paclitaxel may have therapeutic benefits for SSc via modulating TGFβ signaling.

## Abstract 2

In a mouse model of Systemic sclerosis low-dose paclitaxel can suppress an important disease-related pathway, that involves TGFβ and Smad, and lessen fibrosis .

## Introduction

Systemic sclerosis (SSc) is an autoimmune disease characterized by excessive deposition of extracellular matrix (ECM) proteins and obliterative vascular lesions in the skin and internal organs [1]. Although the causative factors of this disease remain to be characterized, the main pathobiological features of SSc comprise three interactive components: autoimmune attack, vascular damage, and a lesion in fibroblasts [2,3]. The fibrotic process consists of massive deposition of connective tissue, mostly collagens, which is frequently responsible for the failure of many organs in patients with SSc. Consequently, an array of antifibrotic agents has been developed to: (1) reduce synthesis, excretion, or polymerization of collagen fibrils; (2) enhance collagenase activity; and (3) neutralize cytokines capable of stimulating collagen synthesis, such as TGFβ (transforming growth factor-beta), interleukin-4, and interleukin-6. To date, several of these antifibrotic agents have been tested in clinical trials [4]. These include *D-*penicillamine, colchicine, interferon gamma, and relaxin. Unfortunately, none of these medicines have any proven efficacy in retarding the fibrotic process [5].

TGFβ is a multifunctional regulatory cytokine that is involved in a large number of cellular activities. TGFβ induces matrix accumulation and tissue fibrosis associated with SSc [4]. In addition, TGFβ promotes endothelial cell apoptosis (a property that may be amplified by the presence of anti-endothelial cell autoantibodies found in SSc), and inhibits smooth muscle cell apoptosis. It also regulates T lymphocyte–mediated immune reactions [6]. Thus, TGFβ is believed to play a central role in the pathogenesis of SSc by activating tissue fibroblasts directly or indirectly through endothelial cells, by regulating lymphocyte function, and by affecting endothelial and smooth muscle cell survival and thrombus formation. Therefore, it is conceivable that inhibition of the aberrant TGFβ signaling may be a promising therapeutic strategy for SSc.

TGFβ initiates its diverse cellular responses by binding to and activating specific cell-surface receptors that have intrinsic serine/threonine kinase activity. The activated TGFβ receptors stimulate the phosphorylation of receptor-regulated Smad2 and Smad3 proteins (R-Smads), which in turn form complexes with Smad4 that accumulate in the nucleus and regulate the transcription of target genes. Inhibitory Smad7 acts in an opposing manner to the R-Smads, inhibiting TGFβ signaling [7]. We have previously demonstrated that endogenous Smad-2, −3, and −4 bind microtubules (MTs) in several cell lines, and the binding provides a negative regulatory mechanism to control TGFβ activity. Disruption of the MT network by chemical agents, such as nocodazole and colchicine, leads to ligand-independent Smad nuclear accumulation and transcription of TGFβ-responsive genes, and increases TGFβ-induced Smad activity [8]. We have also found that inhibitory Smad7 is selectively decreased, and we and others have shown that R-Smad3 is increased, in SSc skin fibroblasts, resulting in uncontrolled TGFβ activity that may be, at least in part, responsible for the aberrant ECM deposition observed in SSc [9]. Indeed, in a hybrid human–severe combined immunodeficient (SCID) mouse skin xenotransplant model, we were able to maintain/reconstitute the SSc phenotype using skin biopsies from SSc patients, indicating that the altered balance between inhibitory Smad7 and R-Smads, due to Smad7 deficiency and Smad3 up-regulation, may represent an intrinsic and persistent defect in tissue fibroblasts that can maintain or even induce SSc lesions autonomously in the absence of altered circulatory or systemic factors [9]*.* The aim of this study was to determine if MT stabilization with low-dose paclitaxel could inhibit TGFβ/Smad signaling, ameliorating the fibrotic changes associated with SSc.

## Methods

### Participant Characteristics

Skin biopsy (6-mm punch) was obtained in an area above the elbow considered to have grossly intact skin thickness as determined by clinical palpation of patients with SSc and in the same location of control participants. Thirty-two patients, of whom 26 were female and 6 were male, aged 38–64 y of age (average 48 y) with diffuse cutaneous SSc were studied. The median duration of skin disease was 5.5 y (2–10 y). Concomitant treatment of SSc patients included the immunosuppressant mycophenolate mofetil, angiotensin-converting enzyme inhibitors, calcium channel blockers, and proton-pump inhibitors. These patients were recruited from the Johns Hopkins University Scleroderma Center. All patients met the American College of Rheumatology criteria for the diagnosis of SSc [10]. Patients with overlap syndromes (e.g., lupus) were excluded. Twelve normal participants, including nine women and three men with an average age of 44 y (range 33–58 y) were also analyzed. All patients and volunteers gave their informed consent, and the study was approved by the Johns Hopkins University Human Subjects Institutional Review Board Committee.

### Skin Transplantation

SCID mice (C.B-17/lcrHsd-scid) were purchased from Jackson Laboratory (Bar Harbor, Maine, United States). Mice aged 6–8 wk and weighing 18–22 g were used for transplantation. Briefly, the skin biopsy patch was trimmed into an oval shape depleted of fat tissue, and placed in 2 mM paclitaxel (Taxol; Sigma, St. Louis, Missouri, United States) or in PBS for 30 min at 4 °C. The tissue was rinsed with PBS just before transplantation. On the dorsolateral surface of the recipient SCID mouse, an oval graft bed approximately 6 mm in diameter was created to fit the graft, leaving the deep fascia layer intact. The trimmed skin patch was transplanted onto the graft bed by suturing the skin patch into the defect with 8–0 suture around the margin of the patch. A total of 25 transplants, including 17 SSc grafts and eight normal grafts, were included in the study (Table 1). The grafts, together with a small ring of the native skin, were harvested at 30 d following transplantation. Nineteen non-transplanted biopsy samples (15 SSc and four normal) were included as nonsurgical controls. Upon sacrifice, the underside of the skin was photographed for angiogenesis study, and the tissue was then divided into two segments: One segment was fixed in 10% neutral buffered formalin and embedded in paraffin, and the other segment was stored in RNAlater (Ambion, Austin, Texas, United States) for RNA expression studies. All animals were cared for in compliance with the “Principles of Laboratory Animal Care and the Guide for the Care and Use of Laboratory Animals,” prepared by the Institute of Laboratory Animal Resources and published by the National Institutes of Health (NO86 to 23, revised 1985).

**Table 1:**
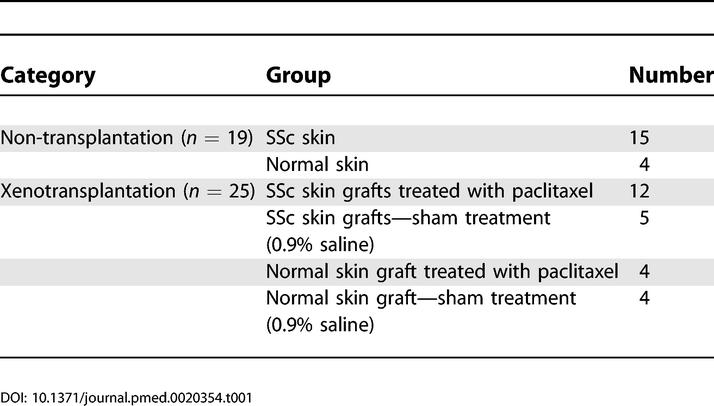
The Summary of Skin Samples Used in the Study

### Immunohistochemistry and Histology

SSc and normal specimens were processed by 10% formalin-fixation and paraffin-embedding. Immunohistochemistry (IHC) for Smad2, Smad3 (Santa Cruz Biotechnology, Santa Cruz, California, United States), phospho-Smad2 (Upstate Biotechnology, Lake Placid, New York, United States), phospho-Smad3 (a generous gift from Dr. Peter ten Dijke, Leiden University, Leiden, The Netherlands), collagen-1 (Santa Cruz Biotechnology), and PAI-1 (American Diagnostica, Greenwich, Connecticut, United States) was performed using Vector's ABC kits (Vector Laboratories, Burlingame, California, United States) on 3-μm consecutive serial sections. Briefly, after deparaffinization, slides were quenched in 3% H_2_O_2_ for 10 min to block endogenous peroxidase and washed in PBS. Sections were incubated with the primary antibody for 1 h and then with biotinylated secondary antibody followed by ABC reagents. Color development was achieved by incubating diaminobenzidine (DAB) as a substrate. Slides were counterstained with Mayer's hematoxylin. Preincubation of the primary antibody with specific blocking peptides or substitution of the primary antibody with an irrelevant IgG served as negative controls. Smad2- and Smad3-positive cells were counted in at least six high-power fields in each sample by two independent observers (CMD and XL). A minimum of 500 cells was counted. Percent positive cells were calculated as the number of positive cells/total number of cells × 100. Cells positive for phospho-Smad2 and phospho-Smad3 were counted in a similar fashion. Among the Smad2- and Smad3-positive cells, the percentage of those stained for phospho-Smad2 and phospho-Smad3 was calculated as the number of phospho-Smad2– and phospho-Smad3–positive cells/the number of cells positive for Smad2 and Smad3, respectively, × 100.

Sections in series with IHC were stained with H & E (hematoxylin/eosin), and Verhoeff's van Gieson elastin and Mason trichrome (VVM). Each section was examined for the presence, extent, and distribution of collagen, elastic fibers, and other matrix proteins.

### Angiogenesis Assessment

The number of microvessels was counted from three to five randomly selected high-power fields (40× magnification) in histology slides stained with H & E. Neovessel formation was also evaluated macroscopically by counting the number of vessels on the underside of the grafts and skin biopsies in a 6-mm field—the entire graft. Angiogenic activity was compared among different groups, including SSc skin biopsy, SSc grafts, normal skin biopsy, and normal skin grafts.

### TaqMan Real-Time Reverse Transcription-PCR

RNA was isolated from the skin using RNeasy Mini kit (Quiagen, Chatsworth, California, United States). One μg total RNA was used for the synthesis of first strand cDNA using the SUPERSCRIPT Preamplification System (Life Technologies, Rockville, Maryland, United States). PCR was optimized for the quantitation of alpha2(I) collagen (*COL1A2;* mouse and human share the same sequence), human *PECAM-1* (platelet endothelial cell adhesion molecule-1), and mouse *PECAM-1* with specific primers and probes. A sequence detector (ABI Prism 7700, PE Applied Biosystems, Foster City, California, United States) was used to measure the amplified product in direct proportion to the increase in fluorescence emission continuously during the PCR amplification. For each sample, a threshold cycle (Ct) value was calculated from each amplification plot, representing the PCR cycle number at which the fluorescence was detectable above an arbitrary threshold. To normalize Ct of the target gene copies to 18S rRNA, ΔCt was calculated as Ct (target) − Ct (18S rRNA). For each sample, the level of *COL1A2,* human *PECAM-1,* and mouse *PECAM-1* was calculated as 2^−ΔCt^. Each sample was tested in triplicate and repeated twice.

### Statistical Analysis

Data are presented as mean ± SEM. Analysis of variance was performed to compare differences among different groups. A *p*-value < 0.05 was considered statistically significant.

## Results

### Paclitaxel Inhibits TGFβ/Smad Signaling and Collagen Deposition in SSc Grafts

**Figure 1:**
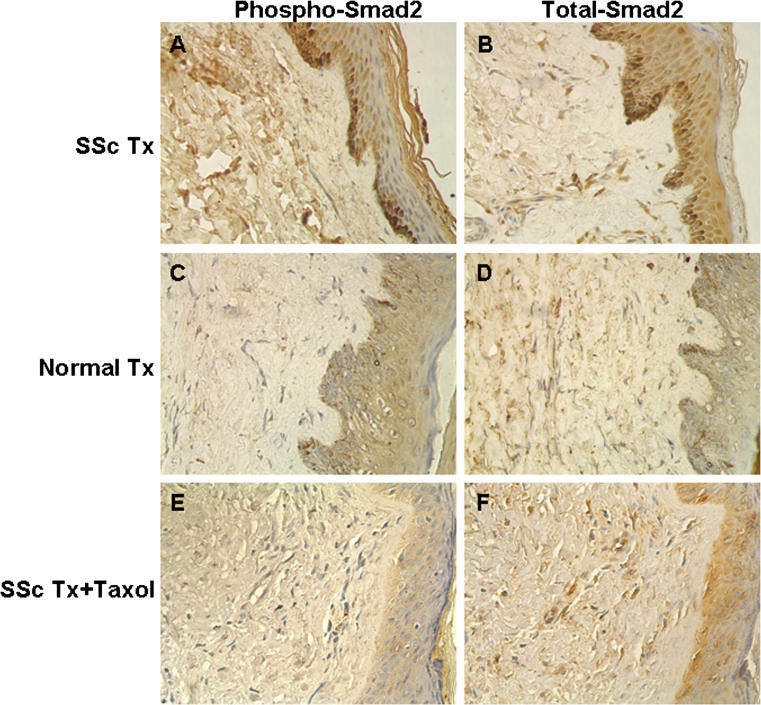
Paclitaxel Treatment Suppresses Smad2 Phosphorylation Strong nuclear staining for phospho-Smad2 (A) and total Smad2 (B) is observed in an SSc skin graft. Smad2 phosphorylation (C) is rare in normal graft, which expresses abundant Smad2 (D). Smad2 phosphorylation is suppressed with paclitaxel (Taxol) treatment (E), without affecting total Smad2 (F) in SSc skin graft. Tx, transplantation.

We demonstrated previously that because of the Smad7 deficiency and increased Smad3 expression in SSc fibroblasts, transplantation of SSc skin retained the SSc phenotype in SCID mice, indicating that the altered balance between positive and inhibitory Smads may represent an intrinsic defect in tissue fibroblasts that can maintain or even induce SSc lesions autonomously in the absence of altered circulatory or systemic factors [9]. Since MT instability enhances TGFβ/Smad signaling pathway [8], we reasoned that stabilization of MTs with paclitaxel might dampen the exacerbated TGFβ signaling in the SSC grafts and prevent the maintenance/reconstitution of SSc phenotype. IHC using anti-phospho-Smad2 antibody revealed enhanced Smad2 phosphorylation, a marker for TGFβ signaling, in SSc tissue, relative to normal skin specimens (87% ± 11% versus 23% ± 15%, *p* < 0.01), when both pre- and post-transplant tissues were analyzed together, whereas the level of total Smad2 remained comparable between normal versus SSc skin tissue. Remarkably, pre-transplant incubation of SSc skin with paclitaxel substantially suppressed the level of Smad2 phosphorylation, approaching that of normal grafts (28% ± 19% versus 21% ± 13%, *p* > 0.05; (Figure1A–1F). Minimal, if any, effects of paclitaxel on Smad2 activity in normal skin grafts were detected (normal grafts + paclitaxel, 26% ± 18% versus normal grafts, 21% ± 13%, *p* > 0.05). Furthermore, the level of total Smad2 and Smad7 (data not shown) remained unchanged.

A similar increase in Smad3 phosphorylation was detected in SSc specimens compared to normal skin tissue (83% ± 11% versus 20% ± 15%, *p* < 0.01). In addition, the level of total Smad3 was increased in SSc versus normal skin tissue (81.5% ± 10% versus 55% ± 21%, *p* < 0.01)—an observation that is consistent with our previous study. Importantly, pre-transplant incubation of SSc skin substantially suppressed the level of Smad3 phosphorylation to that of normal grafts (27% ± 14% versus 24% ± 13%, *p* > 0.05). Furthermore, the expression level of total Smad3 was also downregulated by paclitaxel treatment in SSc (81.5% ± 10% versus 57. 5% ± 12%, *p* = 0.05) (Figure S1A–S1F). There was no detectable effect of paclitaxel on Smad3 activation in normal skin grafts (30% ± 16% versus 25% ± 11%, *p* > 0.05). These findings indicate that preincubation of SSc skin with paclitaxel can effectively offset Smad7 deficiency and Smad3 up-regulation–induced augmented TGFβ signaling in SSc skin grafts, without affecting the total Smad2 and Smad7 expression level. Moreover, when TGFβ signaling is not perturbed in normal skin grafts, paclitaxel does not exert detectable effects.

**Figure 2:**
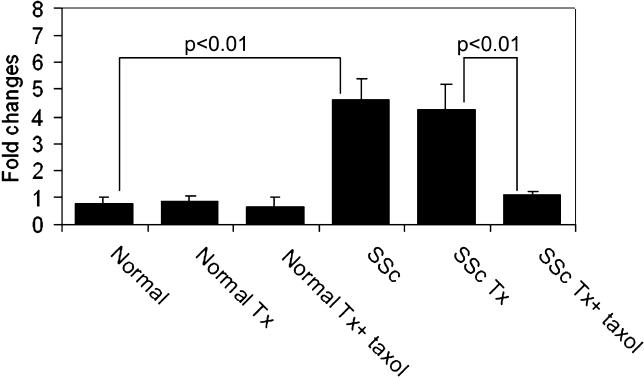
Paclitaxel Decreases COLA2 mRNA Expression in SSc Grafts TRT-PCR analysis shows that the expression of *COL1A2* is reduced 4.5-fold with paclitaxel (Taxol) treatment in SSC grafts, reaching a level equivalent to that of normal skin grafts. By contrast, paclitaxel has no effects on *COL1A2* expression in normal skin grafts.

The progressive accumulation of ECM in the skin and internal organs is a hallmark of SSc, of which collagen type I is the major constituent. Indeed, collagen type I metabolites have been used as markers to evaluate disease activity in SSc [11]. To examine whether paclitaxel treatment affected collagen deposition in SSc skin grafts, we performed quantitative TaqMan real-time reverse transcription-PCR (TRT-PCR) for *COL1A2.* Instructively, the expression of *COL1A2*—a gene whose promoter contains multiple Smad-binding elements (SBE)—was reduced by 4.5-fold (*p* < 0.01) with paclitaxel treatment in SSC grafts, reaching a level that approximated *COL1A2* mRNA expression in normal skin grafts. By contrast, paclitaxel had hardly any effects on *COL1A2* mRNA expression in normal skin grafts (Figure 2).

**Figure 3:**
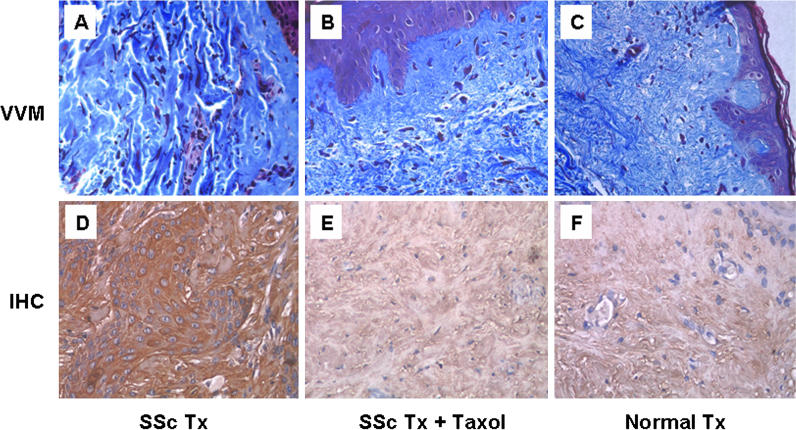
Paclitaxel Decreases Collagen Deposition in SSc Grafts VVM staining shows abundant, thick collagen bundles in the SSc graft (A). The amount of collagen is markedly reduced, and the collagen fibers become finer with paclitaxel (Taxol) treatment (B), comparable to that seen in the normal skin graft (C). Similarly, IHC staining demonstrates intense collagen-1 staining in the SSc graft (D), which is substantially decreased with paclitaxel treatment (E) to a level similar to that seen in the normal skin graft (F).

To confirm the TRT-PCR findings, we performed histological VVM staining to evaluate total collagen deposition and IHC to examine collagen-1 expression level. As shown in Figure 3, there was extensive deposition of total collagen and other ECM proteins, including elastic fibers, in the entire dermis of SSc grafts, which was markedly reduced with paclitaxel treatment. Strong, intense collagen-1 staining was demonstrated in untreated SSc grafts, relative to much weaker staining in paclitaxel-treated SSc tissue, which was comparable to normal skin grafts treated with and without paclitaxel (Figure 3). These data indicate that low-dose paclitaxel prevents the maintenance/reconstitution of the SSc phenotype in SCID mice, and this effect may be mediated by stabilizing MT-Smad complex and subsequent inhibition of TGFβ/Smad signaling.

### Paclitaxel Does Not Affect the Enhanced Local Angiogenesis in SSc Skin Grafts

**Figure 4:**
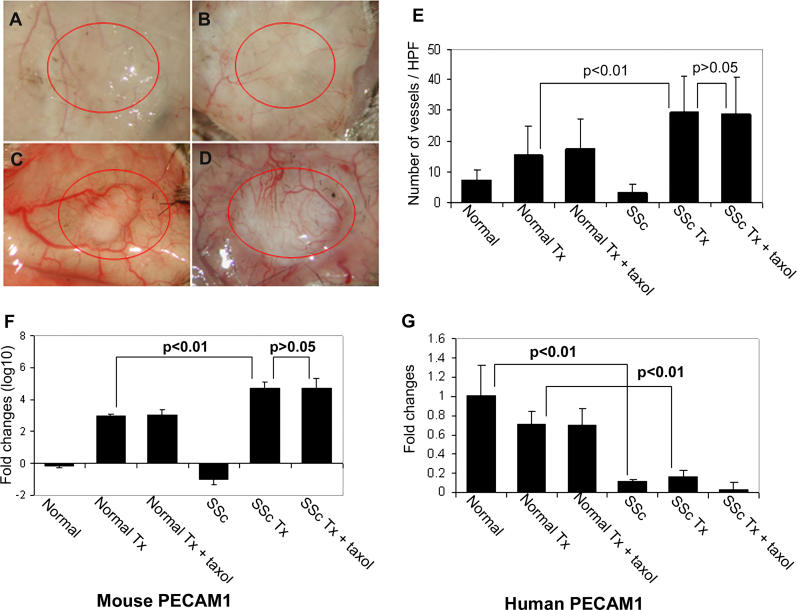
SSc Skin Grafting Stimulates Angiogenesis in SCID Mice Macroscopic analysis of the vessels in the underside of normal (A and B) and SSc (C and D) skin grafts reveals more pronounced angiogenesis in SSc grafts; paclitaxel (Taxol) treatment has no effect on angiogenesis at the macroscopic level (B and D versus A and C). Microscopic analysis shows that the number of vessels in three high-power fields of the SSc grafts is greater than that of normal skin grafts; paclitaxel has no effect on angiogenesis at the microscopic level (E). TRT-PCR for mouse *PECAM-1* demonstrates a substantial increase in mouse *PECAM-1* mRNA expression in SSc and normal skin grafts regardless of paclitaxel treatment; the amplitude of the increase is significantly greater in SSc than in normal skin grafts (F). Human *PECAM-1* is expressed in much lower levels in SSc than in normal skin tissues, indicative of vascular deficiency in SSc; the lack of difference in the expression level of human *PECAM-1* in SSc and normal skin tissues before and after transplantation indicates that the neovascular cells are derived from the recipients (G).

SSc skin lesions are characterized by obliterative microvascular lesions and decreased capillary density, suggesting excessive endothelial injury and/or a deregulated, insufficient angiogenic response [12]. Three basic mechanisms could account for the defective vascular repair in SSc: (1) lack of signals produced by the skin to recruit progenitor cells from the bone marrow, (2) appropriate skin recruitment signals but failure of the bone marrow to mount an adequate repair process, and (3) appropriate skin recruitment signals and adequate bone marrow endothelial progenitor cell (EPC) supply but excessive destruction of EPCs upon their mobilization by immune system. To determine if the defective angiogenic response might be related to lack of signals produced by the skin to recruit progenitor cells from the bone marrow and whether paclitaxel would adversely affect the angiogenic process in SCID mice, we examined neovessel formation following SSc and normal skin transplantation. The EPC supply from the mouse bone marrow is assumed to be nonlimiting. Macroscopic examination of the underside of the 6-mm skin grafts revealed more pronounced angiogenesis in SSc grafts than normal skin transplants (Figure 4A–4D). There were on average 30 vessels in the SSc graft in three high-power fields (40×), as compared with 15 vessels in normal skin grafts (*p* < 0.01) (Figure 4E). To further confirm these findings and determine the origin of the neovascular cells, we performed TRT-PCR for human and mouse *PECAM-1,* an endothelial cell marker. If bone marrow failure and/or autoimmunity-mediated EPC destruction, but not secretion of mobilizing factors by the skin, represented the major bottleneck for a defect repair process in SSc, an aggressive angiogenic response with mouse progenitor cells and, therefore, increased mouse *PECAM-1,* in the SSc grafts would be expected. As shown in Figure 4F, mouse *PECAM-1* was almost undetectable in SSc and normal skin biopsies before transplantation, reflecting the specificity of the primers and probe for the mouse gene. Substantial increase in mouse *PECAM-1* mRNA expression was observed in SSc and normal skin grafts. The amplitude of the increase in mouse *PECAM-1,* however, was significantly greater in SSc than in normal grafts (*p* < 0.01). Human *PECAM-1* was expressed in much lower levels in SSc than in normal skin tissue (*p* < 0.01) before transplantation, indicative of vascular deficiency in SSc. The level of human *PECAM-1* did not change significantly before and after transplantation (Figure 4G). Collectively, these data indicate that the neovascular cells are of recipient mouse origin and that exhaustion of EPC supply from the patient's bone marrow and/or autoimmunity-mediated targeting and destruction of EPCs after mobilization may contribute to the vascular lesion associated with SSc.

The putative activity of paclitaxel to induce endothelial cell or EPC apoptosis and/or to inhibit the proliferation of these cells with subsequent blockade of angiogenesis represented a potential untoward side effect for the use of paclitaxel in the treatment of SSc. To exclude this possibility, we compared the number of vessels in SSc and normal skin grafts treated with and without paclitaxel. As shown in Figure 4A–4E, treatment with paclitaxel did not adversely affect the angiogenic process. A similar increase in neovessel formation was observed in paclitaxel-treated versus nontreated SSc grafts, as compared with pre-transplantation skin biopsies from the same patients. Furthermore, paclitaxel incubation had little, if any, effect on the expression level of *PECAM-1* mRNA, in particular mouse *PECAM-1,* in SSc and normal skin grafts (Figure 4F and 4G). These findings support the notion that low-dose paclitaxel can be used to modulate TGFβ/Smad signaling and treat the fibrotic lesion, without adversely affecting the vascular component of SSc pathobiology.

## Discussion

In the present study, we found that the autonomous maintenance/reconstitution of the SSc phenotype in the hybrid SCID mouse transplant model was substantially prevented by pre-transplant incubation of the SSc skin with 2 mM paclitaxel (taxol), an MT-stabilizing agent. Furthermore, SSc grafts showed a 2-fold increase in neovessel formation, as compared with normal skin grafts, regardless of paclitaxel treatment. The angiogenic process in SSc grafts was associated with a substantial increase in mouse, but not human, *PECAM-1* expression, pointing the origin of the neovascular cells to the recipient mice, perhaps the bone marrow. The angiogenesis data suggest that the skin of patients with SSc has preserved ability to trigger and support an angiogenic response. Collectively, these findings indicate that low-dose paclitaxel may potentially help keep in check the fibrotic process associated with SSc, without adversely affecting the vascular component of the disease.

Members of the TGFβ superfamily play a central role in fibrosis, contributing to the influx and activation of inflammatory cells and fibroblasts and their subsequent elaboration of ECM [4]. TGFβ propagates its signal mainly via a signal transduction network involving receptor serine/threonine kinases at the cell surface and their substrates, the Smad proteins. Upon phosphorylation and oligomerization, R-Smads move into the nucleus to regulate transcription of target genes [7]. Recent findings indicate that the aberrant ECM synthesis by cultured SSc fibroblasts is due, at least in part, to the constitutively enhanced activation of the TGFβ signaling, which may result from the elevated levels of TGFβ receptor type I, inappropriate overexpression/activation of Smad2 and Smad3, and/or decreased Smad7 expression. Indeed, evidence obtained from Smad3-deficient mice shows that TGFβ-induced pro-fibrotic activities are mainly mediated by Smad3 [13]. Smad3-deficient inflammatory cells and fibroblasts do not respond to the chemotactic effects of TGFβ and do not auto-activate TGFβ [14,15]. Furthermore, Smad3-deficient mice are resistant to radiation-induced cutaneous fibrosis, bleomycin-induced pulmonary fibrosis, carbon tetrachloride–induced hepatic fibrosis, and glomerular fibrosis induced by type 1 diabetes caused by streptozotocin [16,17]. We have demonstrated that Smad7, the inhibitory Smad specific for TGFβ signaling, is selectively decreased, whereas Smad3 expression is increased in SSc fibroblasts. TGFβ signaling events, including phosphorylation of Smad2 and Smad3 and transcription of *PAI-1* gene, are increased in SSc fibroblasts, relative to normal fibroblasts. Importantly, the imbalance between Smad7 and Smad3 itself can maintain or induce the SSc phenotype in SCID mice [9]. Furthermore, we have previously shown that MTs serve as a negative regulator for TGFβ/Smad signaling by forming a complex with endogenous Smad2, Smad3, and Smad4, sequestering the R-Smads away from the TGFβ receptor in several cell types [8]. Stabilization of MTs by low-dose paclitaxel can dampen to a normal level the exacerbated TGFβ signaling due to MT instability and block TGFβ-induced inhibition of myogenesis in C2C12 myoblasts [18]. In the present study, we provide evidence indicating that transient incubation of SSc skin with paclitaxel before transplantation into SCID mice substantially suppressed the phosphorylation of Smad2 and Smad3, two homologous Smad proteins that transduce signals from TGFβ and activin. Remarkably, paclitaxel treatment efficiently blocks the autonomous reconstitution and maintenance of the SSc phenotype in SCID mice. These data are consistent with our previous observations, supporting the notion that TGFβ/Smad signaling is regulated by the dynamic stability of MTs, which is sensitive to low-dose MT stabilizing agents, like paclitaxel.

Prolonged chemotherapeutic treatment with paclitaxel has been associated with scleroderma-like changes, albeit in only a small fraction of patients. It is noteworthy that the anti-tumor effect of paclitaxel is mediated via the inhibition of cell proliferation and requires a much higher dosage. The inhibition of TGFβ/Smad signaling, however, can be achieved with very-low-dose paclitaxel. We and others have demonstrated that low-dose paclitaxel had minimal, if any, detectable effects on cell proliferation and other cellular activities, including fibrosis. Intriguingly, low-dose paclitaxel has been shown to inhibit collagen-induced arthritis and other autoimmune disorders in various animal models[19,20]. The low-dose paclitaxel treatment in our human–SCID mouse skin transplant model resulted in marked inhibition of TGFβ/Smad signaling, as evidenced by the decreased phosphorylation of Smad2 and Smad3, and lessened fibrosis. Our data indicate that under our experimental conditions in the SCID mouse, low-dose paclitaxel does not induce scleroderma skin changes. This does not, however, refute the potential linkage between higher doses of paclitaxel used for cancer therapy in humans and skin fibrosis.

The structural and functional vascular and microvascular abnormalities, including Raynaud's phenomenon, represent one of the most important pathological features of SSc [12]. Indeed, microvascular damage and consequent loss of blood supply is found in all involved organs and leads to underperfusion and chronic ischemia, which may play an important role in organ dysfunction and even in the pathogenesis of in SSc. The fibrotic changes may represent a default pathway resulting from vascular failure. Endothelial apoptosis caused by viral infection, immune reactions to viral or environmental factors, reperfusion injury, or anti-endothelial antibodies, is considered a precipitating event in the genesis of vascular lesions in SSc [21]. Recent evidence, however, indicates that vascular repair, particularly that mediated via adult stem cells/EPCs from the bone marrow, plays an important role in maintaining vascular homeostasis and angiogenesis in a variety of disease states [22,23]. It is hypothesized that the vessel wall can deal fairly well with multiple circulating and local noxious stimuli as long as the bone marrow–derived repair capacity remains intact [24]. Indeed, many autoimmune processes might target the repair pathways that are needed to maintain the homeostasis of involved tissues [25]. Adequate vascular repair entails adequate supply of competent progenitor cells in, and their efficient mobilization from the bone marrow, as well as the effective homing to, and subsequent differentiation of these progenitor cells within the vessel wall. Any dysregulation in these processes can tilt the balance of vascular repair and injury in favor of injury and vascular lesion formation. It was postulated that the inadequate angiogenic response in SSc was due to reduced expression of angiogenic factors, such as vascular endothelial growth factor (VEGF), and their receptors. It was recently shown, however, that both VEGF and its receptors (VEGFR1 and VEGFR2) were up-regulated in SSc skin specimens compared with healthy controls [26]. In addition, VEGF protein was significantly increased in blood samples from patients with SSc, reaching levels observed in patients with numerous malignant diseases [27]. Thus, there appears to exist a proper, if not increased, activation of the VEGF/VEGF-receptor axis—key to EPC mobilization—in patients with SSc [26]. One could not, however, rule out the possibility that other, yet to be identified, factors might be missing from the skin and blood of SSc patients, factors that are required to mount and support a successful angiogenic response.

The human skin–SCID mouse transplantation model provides a remarkable opportunity to determine whether lack of signals from the skin of SSc patients to recruit EPCs from the bone marrow might play a role in the vascular lesion formation in SSc, since the bone marrow of the SCID mice is considered intact in terms of EPC supply and there is an absence of immune-mediated destruction of EPCs. Extensive analysis of vascular formation following SSc and normal skin transplantation both at microscopic and macroscopic levels revealed a robust angiogenic response in the SSc grafts, at least twice that seen in normal skin grafts. TRT-PCR for human and mouse *PECAM-1* demonstrated a substantial increase in mouse *PECAM-1* mRNA expression in SSc and normal skin grafts. Furthermore, the amplitude of the increase in mouse *PECAM-1* was significantly greater in SSc than in normal grafts. In contrast, human *PECAM-1* was expressed in much lower levels in SSc than in normal skin tissues before transplantation, indicative of vascular deficiency in SSc, and the expression level of human *PECAM-1* did not change before and after transplantation in SSc and normal skin tissues, indicating that the neovascular cells are derived from the mouse recipients rather than the human donors. These data indicate that the signals from SSc skin, if anything, are stronger in stimulating the mobilization of EPCs from the bone marrow. Moreover, the robust angiogenic activity observed in SSC relative to normal grafts argues against the possibility that the angiogenic response in the SCID mouse is solely due to a wound-healing effect in response to grafting procedures.

It remains to be determined, however, if the EPC supply in the bone marrow or autoimmunity-induced targeting and destruction of EPCs after their mobilization or both serve as the culprit in undermining the vascular repair process, contributing to vascular lesion formation in SSc. Using an animal model of atherosclerosis, we have demonstrated that exhaustion of selected progenitor cell populations, including EPCs and their supporting cells, a process that is accelerated by risk factors, can lead to the inability of the bone marrow to mount a successful vascular repair process, contributing to the initiation and progression of atherosclerotic vascular lesion formation [22,28]. Circulating EPCs and CD34^+^/KDR^+^ precursor cells were reduced in patients with atherosclerotic coronary artery disease. The reduction of these cells represented significant risk factors for atherosclerosis, even after adjusting for most classic risk factors, including age, sex, hypertension, diabetes, smoking, family history, and low-density lipoprotein cholesterol levels. Furthermore, factors that reduce cardiovascular risk, such as statins or exercise, elevate EPC levels, which contribute to enhanced endothelial repair. Hence, a reduced circulating EPC level has been proposed as a significant risk factor for cardiovascular disease. There is conflicting evidence regarding the level of circulating EPCs in SSc patients. Specifically, Kuwana et al. [25] showed that the levels of circulating EPCs, defined by the expression of CD34, CD133, and VEGFR2, were decreased in SSc patients. By contrast, Del Papa et al. [29] found that circulating EPCs—cells positive for CD34 and CD133—were increased in SSc patients, particularly in the early stages of the disease. Although the discrepancy between these studies might be due to the differing definitions of EPCs and different disease stages of SSc patients, it underscores the idea that further investigation is warranted in delineating the relative contribution of inadequate bone marrow EPC supply and excessive destruction of circulating EPCs to the imbalance between vascular injury and repair in SSc.

The established mechanism that confers the antitumor effects of paclitaxel relates to its antiproliferative and antiangiogenic activity when used at large doses and for a prolonged period of time [30]. Indeed, the putative antiangiogenic effect of paclitaxel represented our primary concern for its use in the treatment of SSc. Remarkably, when paclitaxel- and sham-treated SSc and normal grafts were analyzed for their angiogenic response at microscopic and macroscopic levels, similar numbers of vessels were observed, indicating that low-dose paclitaxel does not affect neovessel formation that is likely mediated via EPCs recruited from the bone marrow.

In conclusion, we have demonstrated that SSc skin treated with low-dose paclitaxel can significantly suppress the exacerbated TGFβ/Smad activity of SSc skin and lessen the fibrotic changes upon transplantation into SCID mice. We have found that transplantation of SSc skin into SCID mice elicits effective angiogenesis—an effect that is significantly stronger than with normal skin grafting. Importantly, the neovascular cells are almost exclusively derived from the recipients, perhaps originating from the mouse bone marrow. These observations should shed light on SSc disease pathogenesis and provide evidence for the development of novel therapeutic strategies. The fact that low-dose paclitaxel suppresses fibrosis without dysregulating angiogenesis suggests that fibrosis might be the result of a “default” pathway that develops autonomy once the SSc tissue becomes depleted of blood vessels.

## Supporting Information

Figure S1 Paclitaxel Treatment Suppresses Smad3 Phosphorylation Strong nuclear staining for phospho-Smad3 (A) and total Smad3 (B) is observed in an SSc skin graft. Smad3 phosphorylation (C) is rare in normal graft, which also expresses a low level of total Smad3 (D). Smad3 phosphorylation and perhaps total Smad 3 are suppressed with paclitaxel (Taxol) treatment in SSc (E and F).
(9.5 MB TIF).

**Patient Summary**

**Background**

Systemic sclerosis or scleroderma (SSc) is the name for a group of progressive diseases, all of which involve the abnormal growth of connective tissue. SSc is triggered when the body's immune system turns against the body, causing abnormal production of collagen that can be limited to the skin or extend to the blood vessels and internal organs.

**Why Was This Study Done?**

Various genes have been suggested as being involved in the production of collagen. One of the key genes in this pathway, and in SSc, is TGFβ, which affects the activity of a number of other genes. A part of a cell's internal structure, known as microtubules, affects TGFβ activity. Previous work has shown that microtubules can be affected by drugs, such as the one in this study, paclitaxel, which stabilizes the microtubules.

**What Did the Researchers Do and Find?**

They looked at the effect of paclitaxel on human skin samples that had been taken from people with SSc, or from people with normal skin, and then transplanted into mice. This transplantation is a good way of studying the effects of drugs in this disease without having to test them directly on people. The researchers found that paclitaxel affected the activity of TGFβ and the related genes, and ultimately decreased the amount of collagen that would usually be found in the skin of people with SSc.

**What Do These Findings Mean?**

These findings suggest that paclitaxel would be worth investigating further as a useful drug in the treatment of people with SSc. One concern, however, is that in people who have been treated with high doses of this drug for other conditions, such as some types of cancer, the opposite effect of that shown here has been found: increased collagen has been produced. Therefore, before it can be certain that this drug is safe to use, further work will need to be done to determine what the different effects of the drug are at high and low doses, and what a safe dose is in humans.

**Where Can I Get More Information Online?**

MedlinePlus has many links to pages of information on SSc:

http://www.nlm.nih.gov/medlineplus/scleroderma.html

The Scleroderma Foundation is a non-profit organization based in the United States that provides information on scleroderma for patients, and supports research:

http://www.scleroderma.org/

The Scleroderma Research Foundation, which helped support this work:

http://www.srfcure.org/

## Abbreviations

Ct: threshold cycle
ECM: extracellular matrix
EPC: endothelial progenitor cell
IHC: immunohistochemistry
MT: microtubule
R-Smad: receptor-regulated Smad
SCID: severe combined immunodeficient
SSc: systemic sclerosis
TRT-PCR: TaqMan Real-Time Reverse Transcription-PCR
VEGF: vascular endothelial growth factor
VVM: Verhoeff's van Gieson elastin and Mason trichrome
